# Causes of Death After Bariatric Surgery: Long-Term Study of 10 Years

**DOI:** 10.1007/s11695-024-07466-0

**Published:** 2024-08-31

**Authors:** Nathaniel Rookes, Oday AL-Asadi, Sashi Yeluri, Peter Vasas, Nehemiah Samuel, Srinivasan Balchandra, Abdulzahra Hussain

**Affiliations:** 1 Medical School, University of Sheffield, Sheffield, UK; 2 Homerton University Hospital, London, UK; 3 Doncaster Royal Infirmary, Doncaster, UK; 4 Alkafeel Medical College, Alkafeel University, Najaf, Iraq

**Keywords:** Body mass index, Glucagon-like peptide 1 receptor antagonist, Laparoscopic adjustable gastric band (LAGB), One anastomosis gastric bypass, Roux-en-Y gastric bypass, Laparoscopic sleeve gastrectomy (LSG), Type 2 diabetes mellitus

## Abstract

**Background:**

There is a lack of up-to-date research addressing the causes of death and predictors of long-term mortality after bariatric surgery.

**Methods:**

This was a single-centre retrospective study. Trust records were used to identify deceased patients and their medical history. The demographic data, comorbidities, cause of death, and time since surgery were retrieved and tabulated. Data was recoded to allow for use in IBM SPSS.

**Results:**

There were 39 deaths amongst 891 patients who underwent bariatric surgery between 15th June 2010 to 18th September 2022. The main cause of death was pneumonia and respiratory causes with 15.4% of the cohort. A history of asthma/COPD had an association with the cause of death (*p* = 0.021). A history of hypertension, ischaemic heart disease (IHD), and smoking were all associated with a higher age at death, whilst a history of IHD was associated with a higher number of days from operation to death. Age at operation and number of comorbidities both correlated with age at death, and multiple linear regression of age at death with age at operation and number of comorbidities as predictors was significant (*p* < 0.001). A Cox regression found age at operation to have a significant effect on survival, with a hazard ratio of 1.063 (95% CI:1.027 to 1.100, *p* < 0.001).

**Conclusion:**

Pneumonia and respiratory causes are the largest causes of long-term mortality after bariatric surgery. The only factor found to have a detrimental effect on all-cause mortality was age at operation which reduced survival. Hypertension, IHD, and smoking are indirect factors that are associated with mortality.

**Graphical Abstract:**

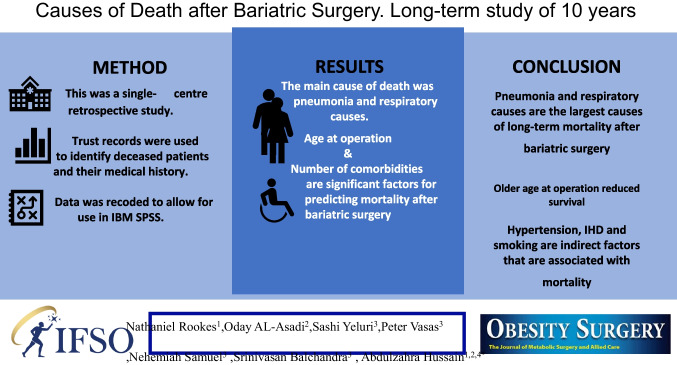

## Introduction

Obesity is a rising global problem. The percentage of adults in England who are classed as individuals with obesity rose by 13.1% from the year 1993 to 2019, whilst those classed as overweight or patients with obesity rose by 11.4% (https://commonslibrary.parliament.uk/). Globally there are over 2.1 billion people who are considered overweight or obese, with around 5% of deaths worldwide caused by a high BMI (www.who.it). Patients being overweight also had links with many of the comorbidities, with T2DM carrying the highest risk for both overweight and patients with obesity groups [1]. The number of patients with obesity in the UK is predicted to rise by 11 million (from 2011 to 2030), increasing the incidence of type 2 diabetes mellitus (T2DM), cardiovascular disease, stroke, and cancer. The cost of treating these comorbidities is estimated to increase by £1.9–2 billion per year in the UK [2]. According to NHS Digital, there were over 1 million admissions where obesity was the primary or secondary diagnosis in 2019/20 [3].

In the UK, obesity has the second largest human-generated economic impact, at $73 billion or 3% of the UK’s GDP [4].

Bariatric and metabolic surgery (BMS) is the most effective tool to control the weight and its associated morbidity. Alongside weight loss, BMS is also effective at inducing remission of many obesity-related comorbidities and can reduce the impact of those comorbidities on patients' lives [5, 6].

General post-operative complications of surgery include events such as anaesthetic, thromboembolic, and cardiorespiratory complications whilst bariatric-specific events include, but not limited, to anastomotic leaks, stenoses, bleeding, with leaks increasing mortality by up to 15% [7].

Despite these difficulties, the peri-operative mortality rates in bariatric surgery are very low. The systematic review of Chang et al. found peri-operative mortality rates (within 30 days of surgery) were between 0.08 and 0.22%, whilst it rose to 0.31–0.35% over 30 days since operation [5]. The review also found that complication rates were between 10 and 17%, and reoperation rates were 6–7%, with the laparoscopic adjustable gastric band (LAGB) having the highest reoperation rates over long-term follow-up [5]. An earlier paper examining the mortality rates between 1995 and 2004, reported a low perioperative mortality of 0.9%, and a total mortality of 2.6% [8]. In the UK, the in‐hospital deathwas (0.07%) and the 30‐day mortality rate after discharge was 0.08% [9], making it safer than other common surgery procedures like knee or hip replacements.

Many bariatric studies focus on weight loss, comorbidities, and 30-day mortality/morbidity, and there is less information about longer-term mortality. Often in these studies, patients are lost to follow-up, potentially concealing long-term patient mortality.

## Materials and Methods

### Aims

The primary aim of this research is to find and examine the causes of death after bariatric metabolic surgery and the factors associated with the cause of death. The secondary aim is to find potential predictors for mortality after BMS.

Hypothesis: The cause of death is associated with one or more patient comorbidities.

### Study Design

This is a single-centre retrospective observational study to examine the long-term all-cause mortality for patients who have undergone bariatric metabolic surgery. The ethics permissions needed for this study were granted by the university of Sheffield ethics committee [050049/18.10.2022].

Data from a local trust was extracted. The inclusion criteria for this study was any patients over the age of 18 who had undergone any surgical bariatric procedure (LAGB, one anastomosis gastric bypass (OAGB), Roux-en-Y gastric bypas (RYGB), or laparoscopic sleeve gastrectomy (LSG)) in our bariatric unit between 15th June 2010 to 18th September 2022. Exclusion criteria: operations that were done outside the above time period and non-primary procedures. The study was sponsored by the university of Shffield as a part of fulfilment for BscMed degree and a copy of full study including the data was submitted to the university.

### Data Collection

Those patients who had died since their operation were identified by searching each patient’s hospital identification number (or National Health Service number where their hospital number was not available) through trust records which provided a date of death if applicable. These searches were made from the 10th to the 13th of February 2023, so any patient that died after these dates would not have been included. To check the correct patient was being searched, the NHS number, date of birth, and sex were cross-referenced between our database and the details given by trust records. The deceased patient’s hospital numbers and NHS numbers were recorded.

Additional information regarding patient demographics and treatment details was obtained via trust records. This additional information included the age (at death) and gender of the patient, the dates of their operation and death, the type of surgery performed, whether this was revised at any point, if they had a history of asthma/COPD, cancer, chronic kidney disease, connective tissue disease, cerebral vascular accident, diabetes, gastro-oesophageal reflux disease, hypertension, ischemic heart disease (IHD), obstructive sleep apnoea (OSA), smoking, alcohol, previous laparotomy, and their cause of death.

Demographical data such as patient hospital number, NHS number, name, sex, age, and date of death were accessible through trust records, whilst information about their operation and medical/surgical history was obtained from letters of correspondence, operation notes, clinic letters and notes, and discharge letters that had been uploaded to the trust’s records. If a patient had any history of being diagnosed with one of the aforementioned medical conditions above, or any history of smoking or alcohol, they were recorded as having that condition/being a smoker/drinker, regardless of whether they had stopped being a smoker/drinker/symptomatic of that disease at the time of death.

### Statistical Analysis

Data was recoded to allow for use in IBM SPSS. Additional variables such as number of days from operation to death and number of comorbidities were calculated.

Descriptive statistics were produced for each variable, mean/median, and standard deviation/interquartile range for continuous variables, and frequencies for categorical variables. The significance level was set at *p* =  < 0.05 for all statistical tests.

Continuous variables were tested for normality of distribution via the Shapiro–Wilk test. Associations between cause of death and history of the factors mentioned previously were examined by Fisher’s Freeman Halton exact test (as Chi-squared assumptions were not met). Significant results then underwent multinomial regression.

To explore potential predictors of mortality, independent samples *t*-test or Mann–Whitney *U*, and one-way ANOVA or Kruskal–Wallis tests were used to investigate whether history of each comorbidity and condition, cause of death, or operation type impacted the age at death, number of days from operation to death, and number of comorbidities. Independent samples *t*-test were used for variables with no more than 2 groups, whilst on way ANOVA was used when a variable had more than 2 groups. Mann–Whitney U and Kruskal–Wallis tests were used when assumptions for independent samples *t*-test and one-way ANOVA were not met. As age at death had an outlier the independent samples *t*-tests were repeated without the outlier included in the analysis.

Correlations between the following were examined (by Pearsons when data was normally distributed and by Spearman’s when data was not normally distributed), age at operation and age at death, age at operation and number of days between operation and death, number of comorbidities and number of days from operation to death, and number of comorbidities and age at death. Simple linear regression was also performed for age at operation and age at death, and number of comorbidities and age at death. Multiple linear regression for age at death by age at operation and number of comorbidities was performed.

Descriptive statistics and survival analysis via cox regression were also performed for the whole cohort, including both alive and deceased patients. The Cox regression included age at operation, sex, and operation type as covariates in the analysis.

All statistical tests were run using IBM SPSS Statistics v28.0.0.0 (190).

## Results

### General Findings

The total number of patients that have undergone bariatric metabolic surgery in our unit between 15th June 2010 and 18th September 2022 was 891. Of those, 39 have since died (4.38%). The mean age at death was 57.33 years (95% CI: 53.78 to 60.88), with the mean number of days from operation to death being 2317.31 days (6.34 years, 95%CI: 1921.21 to 2713.23 days). Of the 39 deceased patients, 66.7% were female, and the whole of the deceased cohort had at least one of the recorded comorbidities, with the median number of comorbidities being 3.

The most common comorbidities were hypertension, OSA, and diabetes with 24 (61.5%), 21 (53.8%), and 20 (51.3%) cases respectively. A history of smoking was more common than a history of alcohol with 24 (61.5%) and 18 (46.2%) cases respectively, and one-third of the patients had undergone previous laparotomy. Amongst the deceased, only 5 (12.8%) patients had their bariatric operation revised.

The most common operation type was RYGB, with 19 of the 39 patients undergoing this operation, followed by LAGB with 11 patients, and then SG and OAGB with 6 and 3 patients respectively.

### Primary Outcomes

The largest cause of death was pneumonia and respiratory causes with 12 deaths, this included 5 deaths caused by COVID pneumonia. The next largest categories were infection/sepsis and liver failure, both with 6 deaths, followed by cardiac causes, malignancy, and multiple organ failure with 5 deaths. The smallest category was cerebrovascular accidents with 2 deaths.

A history of asthma or COPD is not independent of the cause of death (*p*-value = 0.021). History of cerebrovascular accident was close to significance with a *p*-value of 0.053.

### Secondary Outcomes

#### Age at Death Outcomes

Differences in age at death within each categorical variable (history of each comorbidity and condition) were examined by independent samples *t*-test. Those who had a history of hypertension, IHD, or smoking had a significantly higher mean age at death than those without.

Age at operation and age at death had a correlation coefficient of 0.952 and a *p*-value of < 0.001 showing these 2 variables have a strong and significant association.

The number of comorbidities also showed a positive correlation with age at death, with a correlation coefficient of 0.390 with a *p*-value of 0.014.

A multiple linear regression of age at death by age at operation and number of comorbidities produced an adjusted *R* square value of 0.878 and the overall model was significant with *p* < 0.001.

#### Number of Days from Operation to Death Outcomes

The number of days from operation to death was also examined within each categorical variable, with those having a history of IHD having a significantly higher number of days from operation to death than those without, there were no other significant results.

Both age at operation and number of comorbidities showed a slight negative correlation with the number of days from operation to death having coefficients of − 0.176 and -0.177 respectively. Neither of these correlations was significant, however, with age at operation producing a *p*-value of 0.285 and number of comorbidities giving a *p*-value of 0.282.

#### Number of Comorbidities Outcomes

A total of 7 comorbidities had significantly higher mean ranks when a history of the disease is present, including asthma/COPD, malignancy, cerebrovascular accident, diabetes, hypertension, IHD, and OSA.

### Whole Cohort Outcomes and Survival Analysis

Regarding the whole cohort, 74.7% of the patients were female whilst 25.3% were male, RYGB was still the most common at 52.5%, followed by LSG with 25.3%, and OAGB and LAGB with 14.7% and 7.5% respectively. The mean age at operation was 45.51 years (95% CI: 44.80 to 46.22).

**Fig. 1:**
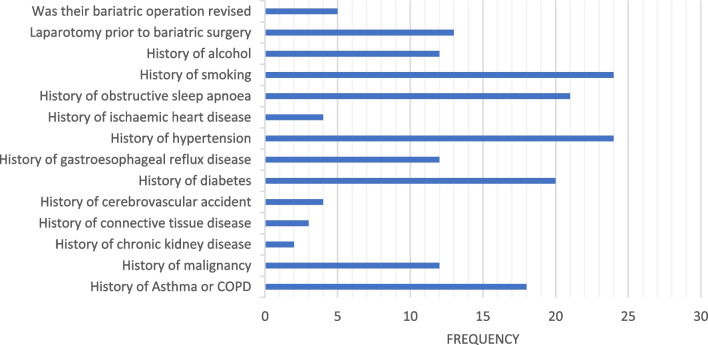
Frequency of each comorbidity

**Fig. 2:**
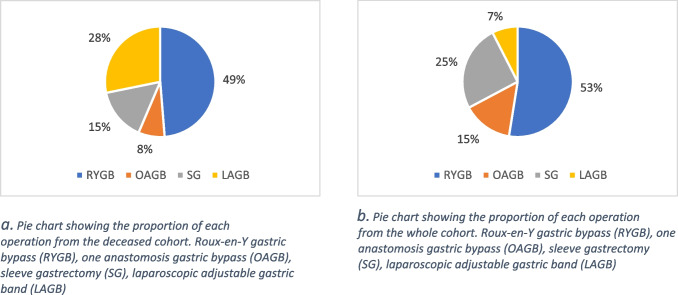
Pie chart showing the proportion of each operation from the deceased cohort. **a** Roux-en-Y gastric bypass (RYGB), one anastomosis gastric bypass (OAGB), sleeve gastrectomy (SG), laparoscopic adjustable gastric band (LAGB). **b** Roux-en-Y gastric bypass (RYGB), one anastomosis gastric bypass (OAGB), sleeve gastrectomy (SG), laparoscopic adjustable gastric band (LAGB)

**Fig. 3:**
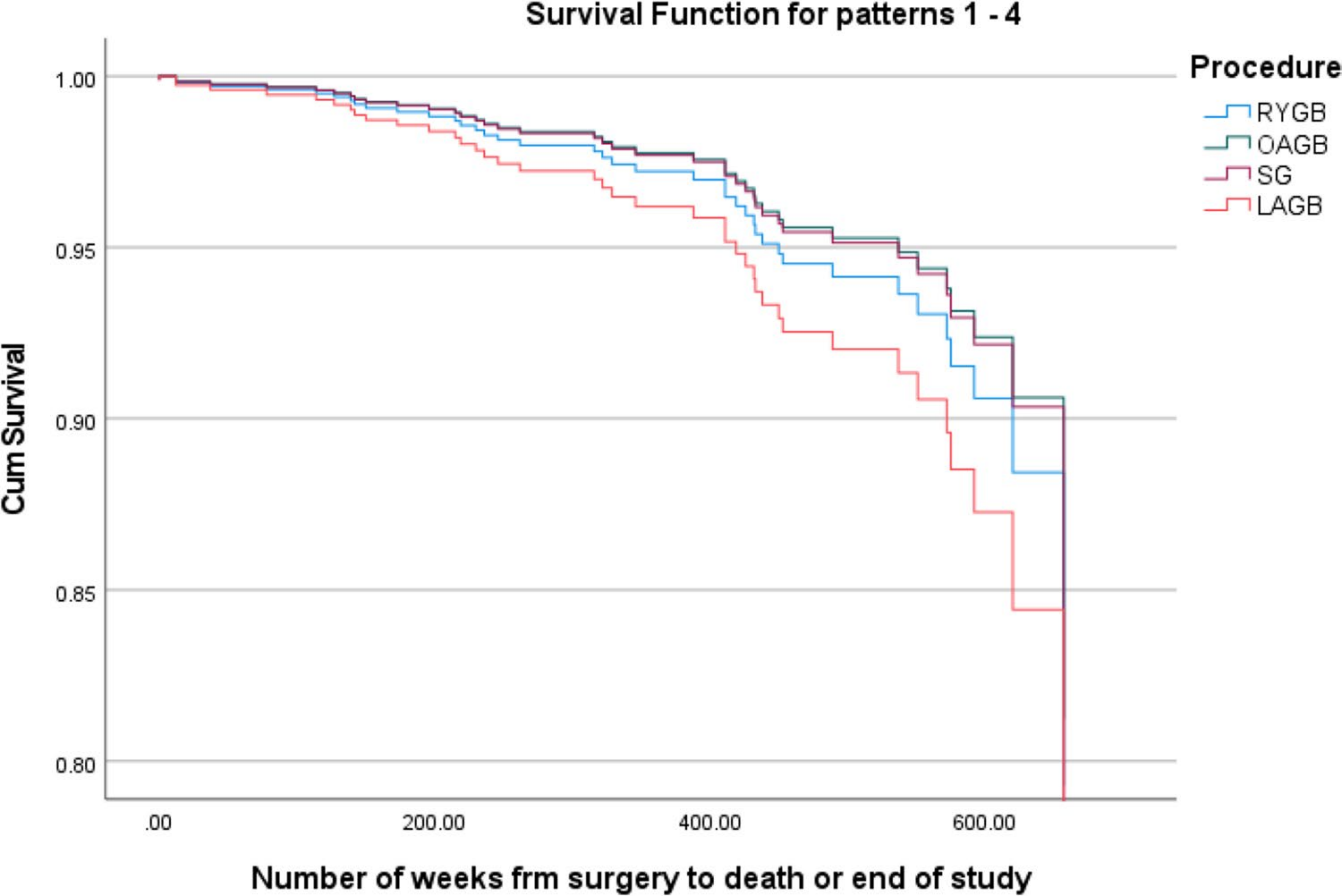
Survival curve by operation type for the whole cohort

**Fig. 4:**
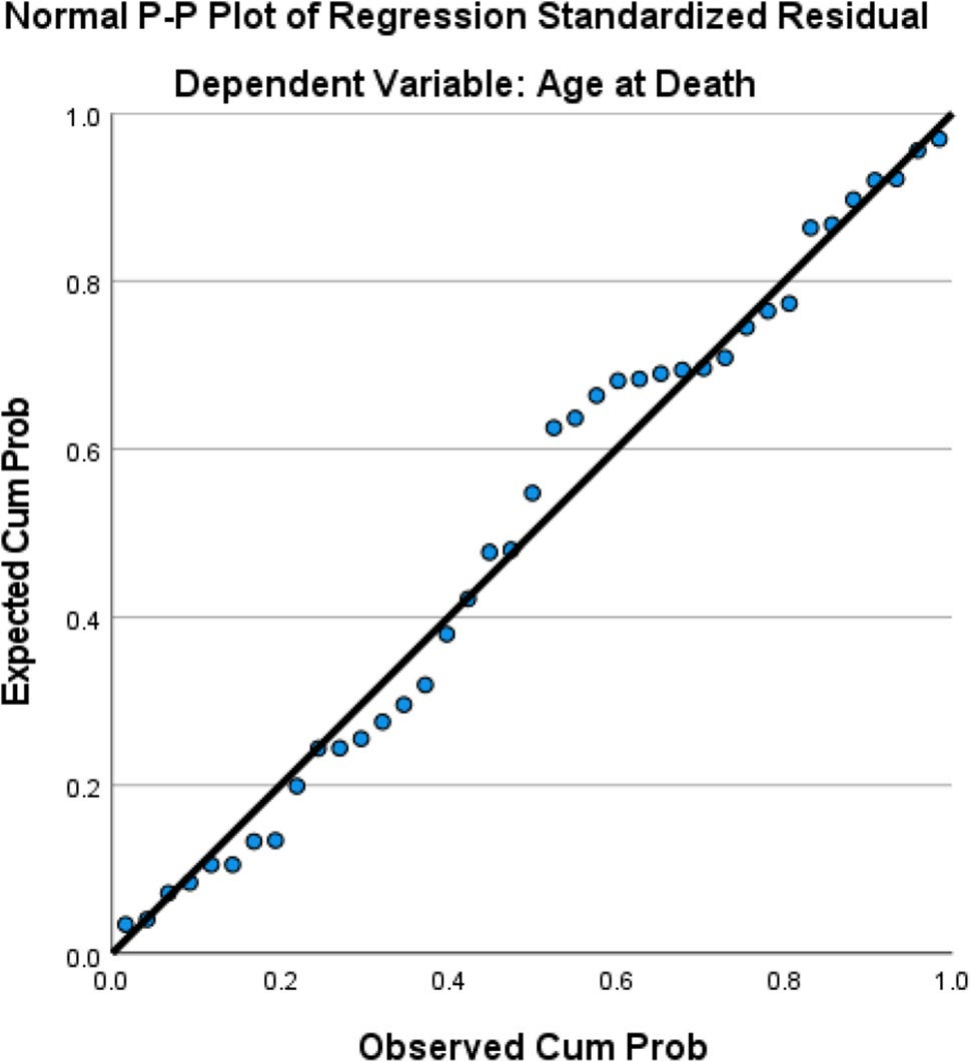
Regression of age at death by number of comorbidities

**Fig. 5:**
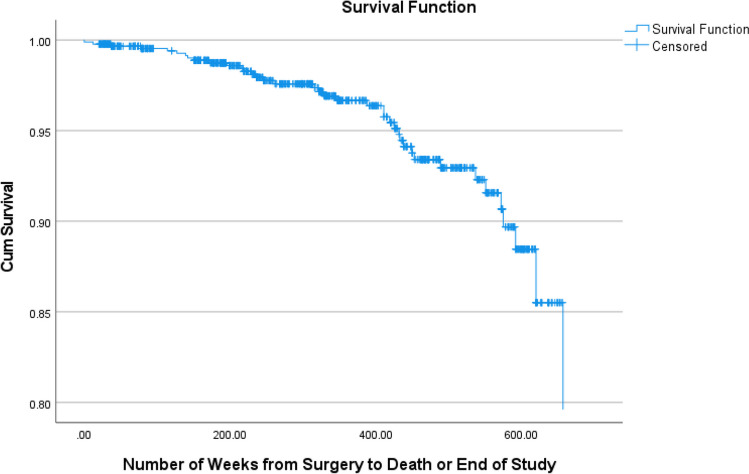
Survival curve for the whole cohort

**Fig. 6:**
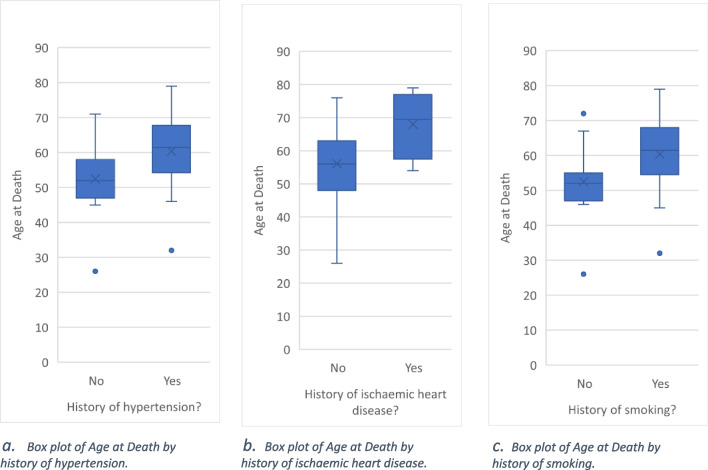
Box plots of age at death and presence of hypertension, ischemic heart disease, and smoking. **a** Box plot of age at death by history of hypertension. **b** Box plot of age at death by history of ischaemic heart disease. **c** Box plot of age at death by history of smoking

The overall analysis had a significant *p*-value of 0.002, meaning the variables included (sex, operation type, and age at operation) do affect survival. The only variable to significantly affect survival was age at operation with a hazard ratio of 1.063. Please see Tables 1, 2, and 3 and Figs. 1, 2, 3, 4, 5, and 6.

**Table 1:** The variables tested for association with cause of death by Fisher’s Freeman Halton Exact test, significance level was 0.05, values given to 3 decimal places

Variable tested	*p*-value
Sex	0.940
History of asthma or COPD	0.021
History of malignancy	0.703
History of chronic kidney disease	1.000
History of connective tissue disease	0.441
History of cerebrovascular accident	0.053
History of diabetes	0.124
History of gastroesophageal reflux disease	1.000
History of hypertension	0.612
History of ischaemic heart disease	1.000
History of obstructive sleep apnoea	0.067
History of smoking	0.702
History of alcohol	0.390
Laparotomy prior to bariatric surgery	0.741
Was their bariatric operation revised	0.965
Type of operation	*0.948*

**Table 2:** Mean and *p*-values generated by independent samples *t*-test and one-way ANOVA comparing mean age at death within each variable. Values have been given to 4 significant figures

Variable		Mean age at death	*p-*value
Sex	Female	56.65	0.602
	Male	58.69	
History of asthma or COPD	No	57.48	0.933
	Yes	57.17	
History of malignancy	No	56.56	0.526
	Yes	59.08	
History of chronic kidney disease	No	56.84	0.244
	Yes	66.50	
History of connective tissue disease	No	56.53	0.124
	Yes	67.00	
History of cerebrovascular accident	No	56.83	0.416
	Yes	61.75	
History of diabetes	No	55.63	0.366
	Yes	58.95	
History of gastroesophageal reflux disease	No	57.26	0.952
	Yes	57.50	
History of hypertension	No	52.47	0.031
	Yes	60.38	
History of ischaemic heart disease	No	56.11	0.045
	Yes	68.00	
History of obstructive sleep apnoea	No	56.39	0.635
	Yes	58.14	
History of smoking	No	52.47	0.031
	Yes	60.38	
History of alcohol	No	59.57	0.185
	Yes	54.72	
Laparotomy prior to bariatric surgery	No	57.04	0.821
	Yes	57.92	
Was their bariatric operation revised	No	56.76	0.420
	Yes	61.20	
Type of operation	RYGB	53.11	0.057
	OAGB	61.33	
	SG	56.00	
	LAGB	64.27	
Cause of death	Cardiac causes	54.00	0.812
	Cerebrovascular accident	68.00	
	Infection/sepsis	65.33	
	Liver failure	56.67	
	Malignancy	60.00	
	Multiple organ failure	45.50	
	Pneumonia and respiratory causes	58.33

**Table 3:** Mean rank and *p*-value generated by Mann–Whitney *U* and Kruskal–Wallis tests used to compare the number of comorbidities within each variable. Values have been given to 4 significant figures

Variable		Mean rank	*p-*value
Sex	Female	19.79	0.867
	Male	20.42	
History of asthma or COPD	No	16.07	0.018
	Yes	18.00	
History of malignancy	No	16.96	0.011
	Yes	26.83	
History of chronic kidney disease	No	19.38	0.136
	Yes	31.50	
History of connective tissue disease	No	19.03	0.060
	Yes	31.67	
History of cerebrovascular accident	No	18.59	0.020
	Yes	32.38	
History of diabetes	No	15.26	0.010
	Yes	24.50	
History of gastroesophageal reflux disease	No	18.94	0.377
	Yes	22.38	
History of hypertension	No	15.07	0.030
	Yes	23.08	
History of ischaemic heart disease	No	18.34	0.006
	Yes	34.50	
History of obstructive sleep apnoea	No	13.58	< 0.001
	Yes	25.50	
History of smoking	No	16.77	0.154
	Yes	22.02	
History of alcohol	No	22.62	0.114
	Yes	16.94	
Laparotomy prior to bariatric surgery	No	20.17	0.891
	Yes	19.65	
Was their bariatric operation revised	No	19.22	0.257
	Yes	25.30	
Type of operation	RYGB	16.55	0.161
	OAGB	28.83	
	SG	19.08	
	LAGB	24.05	
Cause of death	Cardiac causes	6.500	0.180
	Cerebrovascular accident	14.00	
	Infection/sepsis	7.330	
	Liver failure	12.33	
	Malignancy	9.000	
	Multiple organ failure	2.500	
	Pneumonia and Respiratory causes	13.5

## Discussion

### Initial Findings

The total all-cause cumulative mortality rate for those undergoing bariatric metabolic surgery in our unit was 4.38%, with a 30-day mortality rate of 0.11%. This result is comparable with the results of the review performed by Chang et al. which gave 30-day mortality of 0.08–0.22%, although the mortality reported after day 30 was comparatively lower at 0.31–0.35%[5]. The average death rate for the whole UK population is 10.4/1000 population annually [https://www.ons.gov.uk/] with an average 10-year total mortality at 10.4%. This is much higher than the mortality for people who underwent bariatric surgery in our study. According to BOMSS figures [April 2023–March 2024] (National Bariatric Surgery Registry, NBSR,.www.bomss.org), 30-day mortality post-bariatric surgery is 0.04%, which is lower than the 30-day mortality found by this study Table 4.

**Table 4:** The demographic features and intra and post operative complication rates

		Procedure									
		One anastomosis gastric bypass			Roux-en-Y gastric bypass			Sleeve gastrectomy			
		Mean	*n*	Column *N* %	Mean	*n*	Column *N* %	Mean	*n*	Column *N* %	*p*-value
Age		53			49			51			0.098
Preoperative weight		132.87			139.91			137.12			0.525
Preoperative BMI		46.96			48.51			49.15			0.606
Preoperative HbA1c		59			61			61			0.804
complications	intraop		8	34.0%		20	33%		7	31%	0.950
	postop		15	66.0%		40	67%		15	69%	

There is no single study of long-term post-bariatric metabolic surgery mortality in the UK. Two studies on BMS mortality from the USA both reported higher 30-day mortality rates, with a 30-day mortality rate of 1.9% and 0.9%, and longer-term mortality rates of 11.8% (at 15 years) and 2.6% [8–10]. These studies were published in 2004 and 2007 respectively, whereas the first bariatric operation in our unit was in 2010; both these studies were performed in USA with much larger sample sizes, which may explain the differences between the mortality rates. Additionally, the development of skills, infrastructure, medical equipments, pre-operative screening methods, and governance systems possibly impacted the improvement in mortality over the last 15 years.

The mean age at operation for the whole cohort was also similar to previous research at 45.51 years old, with the Chang et al. review and the Omalu et al. study finding the mean age at operation to be 44.6 and 48 years old respectively [5, 8]. The mean age at operation for deceased patients was higher at 50.87.

Bariatric metabolic surgery tends to have a higher proportion of female patients than male, as reflected by the whole cohort being 74.7% female (amongst deceased patients this was 66.7%). Exact figures vary, with some authors reporting 79% of patients being female, whilst a systematic review and meta-analysis looking at surgical and non-surgical treatment of obesity found a lower proportion of female patients at 62.0% [5, 11].

### Primary Outcomes

The largest cause of death in this cohort was pneumonia and respiratory causes, with 31.6% of the known deaths (15.4% when missing cases are included), following this was liver failure and infection/sepsis with 15.8% each of known deaths (7.7% each including missing cases). This contrasts with the paper produced by Omalu et al. which observed much lower rates of pneumonia and lung disease deaths (4.1% and 4.6% of natural deaths respectively), whilst coronary heart disease was the lead cause of death with 19.2% of natural deaths [8]. White et al. pointed out that cardiovascular disease deaths accounted for the most significant factor (36%) of all deaths 5–7 years following bariatric surgery in their 2458-patient series [12]. The difference in causes of death may be explained by different sample size, locations, the COVID-19 pandemic, and by the difference in periods of the studies (1995–2004 and 2010–2022).

One-third of the pneumonia and respiratory deaths were caused by COVID-19 pneumonia, which potentially inflates the number of deaths you may otherwise see in this category. Just 5.3% of deaths (2.6% including missing cases) were caused by cerebrovascular accident which is more comparable to the results found by the Omalu et al. paper, which found that stroke accounted for 3.0% of deaths [8].

Since pneumonia and respiratory causes are the largest causes of long-term mortality after bariatric metabolic surgery, it is important to implement other strategies to manage this condition, e.g., more precise pre-operative lung function tests, post-operative and long-term chest care education, or even precise investigation of the cause of pneumonia and respiratory deterioration. Patients with pre-existing respiratory disease need more attention and the risk of death should be discussed during the pre-operative consultation.

Associations between the cause of death and all collected comorbidities were tested, with only a history of asthma/COPD returning a significant result, showing a history of asthma/COPD is not independent of the cause of death. Due to the small sample size, however, it was not possible to test this association further and determine which cause of death a history of asthma/COPD is most associated with. This association could also have been potentially influenced by COVID-19, as moderate to severe asthma is associated with a higher risk of COVID-19 mortality and a third of pneumonia and respiratory caused deaths in this cohort were due to COVID pneumonia [13].

### Secondary Outcomes

#### Incidence of Comorbidities

Every deceased patient had at least one comorbidity, with the most common comorbidities amongst deceased patients being hypertension, OSA, and diabetes with 61.5%, 53.8%, and 51.3% of the cohort respectively. Rates of hypertension vary, with Wolfe et al. and Osland and Memon finding similar proportions of hypertension in obese patients at around 65% and 66% respectively, whilst the Chang et al. review reported lower rates at 47% [5, 6, 14]. OSA and diabetes are reported at a lower level in the literature, with OSA between 25 and 40%, and T2DM between 25 and 27% [5, 6, 15]. This difference is of interest, as previous research has shown that bariatric surgery is effective at inducing T2DM remission [5, 6, 11, 14–16].

#### Influence of Comorbidities

Bariatric frailty syndrome is associated with higher mortality and morbidity, with the total effects of the existing co-morbidities being higher than if these co-morbidities were simply added [16].The obesity surgery mortality risk score (OS-MRS) after bariatric surgery has been investigated before [17–20]. It is a useful tool, but the validity is not very high in all cases, and focusing mainly the peri-operative risk [21].

Age at death, number of days from operation to death, and number of comorbidities were investigated further by testing whether each one had any association with any of the comorbidities collected, operation type, or cause of death. Comorbidities with a significantly lower age at death or number of days from operation to death may offer a potential predictor of mortality. Significant results for age at death (when outliers were included) were a history of hypertension (*p* = 0.031), a history of IHD (*p* = 0.045), and a history of smoking (*p* = 0.031), with these comorbidities being associated with higher age at death. The only significant result for the number of days from operation to death was having a history of IHD (*p* = 0.030), with the mean number of days from operation to death being higher in the 4 patients who had IHD than the 35 who did not. This suggests that those with a history of hypertension, IHD, or smoking die at an older age than those who do not and those with IHD have a higher number of days from operation to death, however, this analysis does not consider those patients that are still alive that may or may not have a history of those comorbidities.

Several comorbidities also positively correlated with age at death, although the correlation coefficient was much lower at 0.390. This suggests that a higher number of comorbidities is associated with a higher age at death, although again this analysis does not include patients that are still alive.

The Cox regression found age at operation to have a significant effect on survival with a hazard ratio of 1.063. It also showed that sex and operation type do not have a significant effect on survival. This finding is perhaps an expected one given that a 2020 study examining the impact of age on mortality found that those over 45 years of age at the time of operation had higher mortality than those below 45 [22].

## Limitations

This research is limited by the fact that it is both retrospective and observational. In addition, this research was conducted using one location, and as such has a small sample size.

## Conclusions

Bariatric metabolic surgery has low mortality rates, with pneumonia and respiratory causes being the largest long-term cause of mortality, although this category may have been inflated by deaths from COVID-19.

A history of asthma/COPD was significantly associated with all-cause mortality.

Hypertension, IHD, and smoking indirectly affect mortality.

Age at operation does affect overall survival.

The only potential predictor of mortality was age at operation which reduced survival in our patients.
